# Strategies to Circumvent the Side-Effects of Immunotherapy Using Allogeneic CAR-T Cells and Boost Its Efficacy: Results of Recent Clinical Trials

**DOI:** 10.3389/fimmu.2021.780145

**Published:** 2021-12-15

**Authors:** Sergei Smirnov, Alexey Petukhov, Ksenia Levchuk, Sergey Kulemzin, Alena Staliarova, Kirill Lepik, Oleg Shuvalov, Andrey Zaritskey, Alexandra Daks, Olga Fedorova

**Affiliations:** ^1^ Almazov National Medical Research Centre, Personalized Medicine Centre, Saint Petersburg, Russia; ^2^ Institute of Cytology, Laboratory of Gene Expression Regulation, Russian Academy of Sciences, Saint Petersburg, Russia; ^3^ Institute of Molecular and Cellular Biology SB Russian Academy of Science (RAS), Department of Molecular Immunology, Laboratory of Immunogenetics, Novosibirsk, Russia; ^4^ Belarusian Research Center for Pediatric Oncology, Hematology and Immunology, Oncological Department 3, Borovliani, Minsk Region, Belarus; ^5^ RM Gorbacheva Research Institute of Pediatric Oncology, Hematology and Transplantation, Chemotherapy and Bone Marrow Transplantation Department, Saint Petersburg, Russia; ^6^ Pavlov University, Department of Hematology, Transfusiology and Transplantology, Saint Petersburg, Russia

**Keywords:** chimeric, allogeneic, clinical, trials, GvHD, receptor, T cell, CAR (chimeric antigen receptor)

## Abstract

Despite the outstanding results of treatment using autologous chimeric antigen receptor T cells (CAR-T cells) in hematological malignancies, this approach is endowed with several constraints. In particular, profound lymphopenia in some patients and the inability to manufacture products with predefined properties or set of cryopreserved batches of cells directed to different antigens in advance. Allogeneic CAR-T cells have the potential to address these issues but they can cause life-threatening graft-versus-host disease or have shorter persistence due to elimination by the host immune system. Novel strategies to create an “off the shelf” allogeneic product that would circumvent these limitations are an extensive area of research. Here we review CAR-T cell products pioneering an allogeneic approach in clinical trials.

## 1 Introduction

Recently, therapy using chimeric antigen receptor T cells (CAR-T cells) has emerged as a powerful tool for patients with certain subtypes of B-cell lymphoma and leukemia. CAR-T cells are designed to selectively target a predefined tumor-associated antigen. This is achieved by the expression of a chimeric antigen receptor (CAR) on the surface of immune cells, typically T cells or natural killer (NK) cells (1).

CARs are composed of an antigen-binding domain (e.g., a single-chain variable fragment (scFv) derived from a monoclonal antibody) and a signaling domain (e.g., the intracellular portion of the cluster of differentiation (CD)3ζ subunit of the T-cell receptor) that are linked together *via* a transmembrane and an optional hinge domain. This design allows for major histocompatibility complex (MHC) molecule-independent antigen binding, which launches downstream signaling events culminating in a cytotoxic response and destruction of the target cell (2). Second- and third generation CAR-T cells carry additional co-stimulatory domains such as those derived from CD28 or 4-1BB (3). For instance, recent studies have shown that CAR-T cells with a CD28 costimulatory domain exhibit rapid activation of CAR-T cells followed by exhaustion (4, 5). By contrast, incorporation of a 4-1BB-based co-stimulatory domain ensures prolonged endurance of CAR-T cells by diminishing expression of exhaustion-related genes (6), which enhances the capacity for oxidative metabolism and central memory differentiation (7). However, large-cohort clinical data have demonstrated that CAR-T cells with CD28 or 4-1BB costimulatory domains can mediate long-lasting remission and have shown comparable results against B cell lymphomas (8, 9) and acute lymphoblastic leukemia (10–13). Considering many differences except from co-stimulatory domains in trials performed up to date (8–10, 12, 14), an additional trial could clarify the possible superiority of CD28 or 4-1BB domains.

More advanced designs of “armored” CAR-T cells have been developed to modify the immunosuppressive tumor microenvironment, and also to enhance T-cell functioning/trafficking and ameliorate CAR-T cell-associated toxicities (15, 16). For instance, interleukin (IL)-7 has been shown to play an essential part in the antigen-driven expansion of naive and activated T cell populations (17) whereas C–C motif chemokine ligand CCL21 is a chemokine implicated in attracting naive T cells and antigen-presenting cells, and coordinating their interaction and consequent tumor antigen-specific immune response (18). The co-expression of IL-7 and CCL21 along with a CAR has led to significant improvement in the proliferation of CAR-T cells *in vitro* and boosted therapeutic activity *in vivo* (18). Antigen-driven signaling *via* second- and third-generation CARs has been described to induce proliferation of CAR-T cells. Excessive immune stimulation, however, can manifest as increased serum levels of cytokines such as IL-6, interferon-γ, and tumor necrosis factor (TNF). This action may lead to uncontrolled systemic inflammation, and is one of the most frequent side-effects of CAR-T cell therapy, referred to as “cytokine release syndrome” (CRS) (19). The second common adverse effect of therapy using CAR-T cells is known as an “immune effector cell-associated neurotoxicity syndrome” (ICANS), which results from increased cytokine levels and their penetration across the blood–brain barrier (20).

In patients with hematological malignancies, most clinical trials have focused on an autologous approach that utilizes T cells isolated from the patient’s peripheral blood. However, in spite of remarkable clinical outcomes (10, 21, 22), this strategy is endowed with several important limitations, namely, very high treatment costs and individual manufacturing processes (23) with possible issues (24–27), reaching 9% in Kymriah pivotal trial (28). Despite comparable time burdens of autologous and allogeneic manufacturing processes, the key difference is that allogeneic CAR-T cells represent an “off the shelf product” that can be administered without delay, which is very important for patients with highly proliferative diseases such as acute leukemia. In addition, T-cell dysfunction and a reduction in the number of naive and central memory T-cell subsets due to chemotherapy (29) or the tumor microenvironment (30) impair *ex vivo* expansion and the persistence of autologous CAR-T cells (31, 32).

CAR-T cells produced from the material of allogeneic donors have three main advantages compared with therapies using autologous CAR-T cells. First, allogeneic CAR-T cells can be produced in advance and delivered without delay according to the established treatment program of the individual patient. Second, allogeneic donor-derived T cells are not exposed to multiple rounds of anti-leukemia therapy, so they are more amenable to *ex vivo* manipulation (33). Third, products based on autologous CAR-T cells cannot be manufactured 3 for some patients because of their profound lymphopenia, which is not an issue with products based on allogeneic CAR-T cells.

Despite the numerous advantages of therapy using allogeneic CAR-T cells, this technology comes with two major disadvantages. First, the recipient’s cells appear “foreign” to the native T cell receptors (TCRs) of the administered CAR-T cells, which may induce their activation and result in acute graft-versus-host disease (GvHD). Second, CAR-T cells are foreign to the host immune system, which may cause their rapid elimination from the circulation, and a lack of durable persistence of CAR-T cells in turn has been demonstrated to be associated with poor patient responses in an autologous setting (34).

T cells used for the manufacture of allogeneic CAR-T cells are derived mainly from peripheral blood mononuclear cells, particularly cells that have a TCR consisting of an α and β chain (αβ T cell subsets), which constitute ~90% of circulating T cells in healthy donors (35). On the one hand, due to their relative abundance in peripheral blood and ability to proliferate rapidly, αβ T cells represent an attractive target for the manufacture of allogeneic CAR-T cells. On the other hand, this subset of cells has been shown to play a major part in the pathogenesis of acute and chronic GvHD due to an inherent immunologic mismatch between the patient and donor. In autologous settings, the T cells of patients that undergo negative selection in the thymus are used, thereby comprising a population that is non-responsive to self-peptides in a complex with MHC-I molecules (36). By contrast, in allogeneic settings, the administered cells can recognize healthy recipient’s tissues *via* TCRs in an MHC molecule-dependent manner, with subsequent induction of apoptosis of healthy cells and GvHD manifestation (37–39).

The manufacturing processes of allogeneic CAR-T cells comprise all the production steps of the autologous products that have been reviewed elsewhere (40) with additional, more sophisticated gene-editing steps that will not be discussed in detail here. Turtle et al. pointed out that the defined ratio of CD8+ and CD4+ T-cell subsets is essential for the *in vivo* expansion and persistence of CAR-T cells (41). However, CAR-T cells so far have been administered in both defined (41) and undefined (10, 21, 22) CD4/8 ratios without any loss of efficacy. In an autologous setting, the endurance and efficacy of CAR-T cells have been found to correlate with the numbers of less differentiated CD8+ and CD4+ T-cell subsets in the final product (42). In particular, Xu et al. (43) discovered that the expansion of CD19-redirected T cells was dependent upon the frequency of CD8+CD45RA+CCR7+ subsets corresponding to stem cell-like memory and naive T-cell phenotypes. Conversely, patients after previous lines of therapy often suffer from lymphopenia and have higher quantities of effector memory T cells (42): an allogeneic approach could circumvent this issue because the cells are derived from the peripheral blood mononuclear cells of a healthy donor. In addition, in autologous and allogeneic settings, the number of T cells can be amplified in more defined subsets *via* modulation of the culture conditions. Yang et al. showed that supplementing media with IL-15 and IL-7 during *ex vivo* expansion increased the number of naive T cells (43).

Herein, we review relevant clinical data on the use of therapies based on allogeneic CAR-T cells. We discuss the outcomes of strategies aiming to mitigate GvHD and also the other side-effects associated with therapy using allogeneic CAR-T cells described in recent clinical trials.

## 2 Sources of Allogeneic CAR-T Cells

Building upon clinical experience, the ability of allogeneic CAR-T cells to eliminate tumor cells is dependent upon the initial expansion, duration of persistence, absence of GvHD and also on the ability of the host immune system to reject these cells. When devising strategies for administering allogeneic CAR-T cells, different approaches to reduce the risk of GvHD (e.g., selection of T-cell subsets, use of virus-specific memory T cells or gene editing) could be implemented.

### 2.1 Genetically Modified αβ T Cells

**Figure 1:**
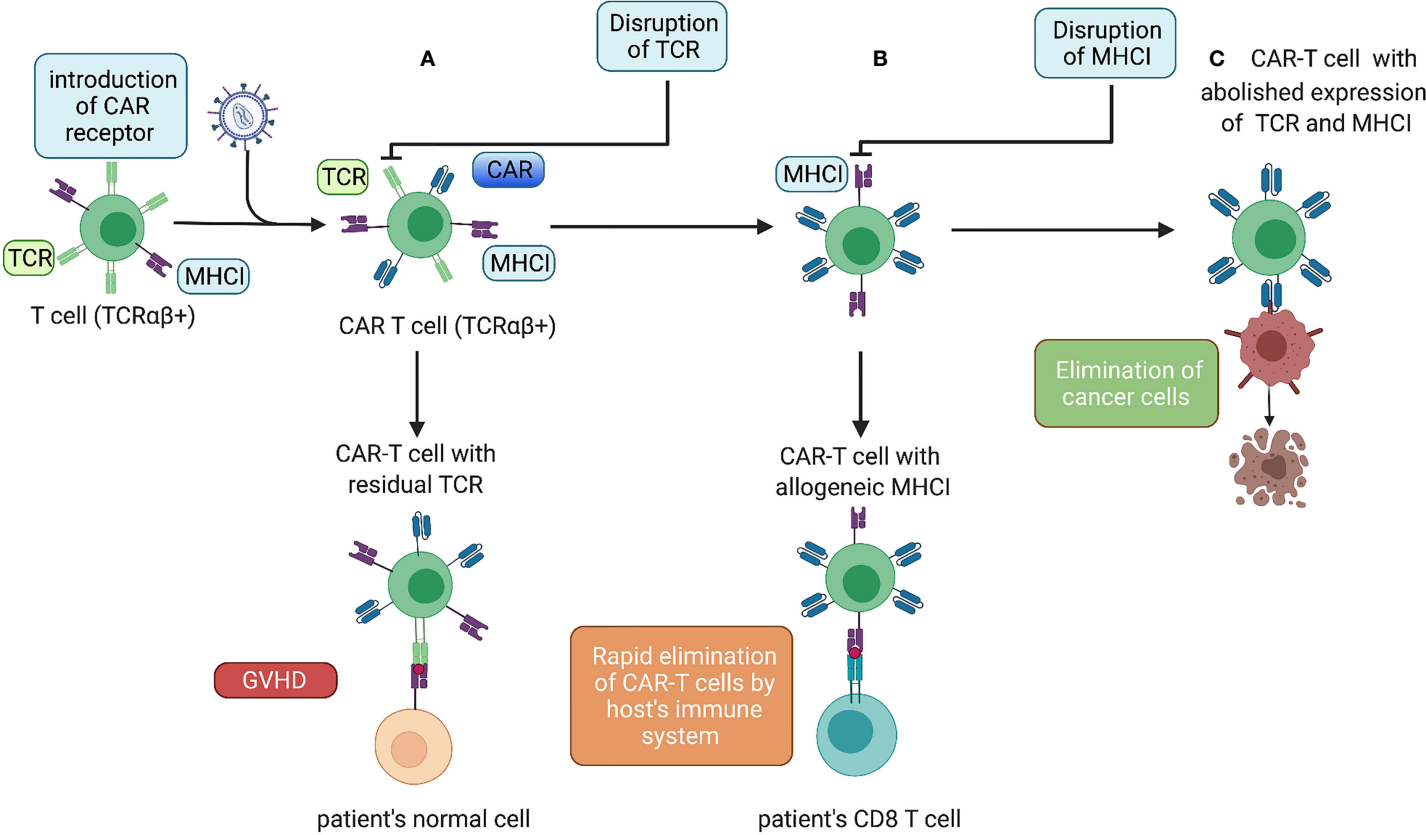
Genetic modifications of allogeneic CAR-T cells. **(A)** Lentiviral transduction of allogeneic T cells with a CAR. Donor CAR-T cells with allogeneic TCRs administered to a patient causing GvHD. **(B)** After an additional gene-editing step to disrupt TCR expression, the cells do not cause GvHD but can be eliminated rapidly by the host immune system. **(C)** The next gene-editing step abolishes expression of MHC-I molecules, thereby ensuring the prolonged endurance and persistence of allogeneic CAR-T cells.

In addition to the introduction of the “CAR-encoding cassette” most commonly delivered by lentiviral and gamma-retroviral vectors (44), we can distinguish two major types of T-cell genetic modifications to obtain allogeneic CAR-T cells with a reduced risk of GvHD and alloimmunization (**Figure 1**).

#### 2.1.1 Disruption of TCRs

The first modification aimed to limit GvHD is the disruption of TCRs. This can be undertaken in three distinct ways: expression of the inhibitory protein(s); by knockout (KO) of the genes encoding TCR chains with site-specific nucleases such as transcription activator-like effector nucleases (TALENS) or clustered regularly interspaced short palindromic repeats/CRISPR associated protein 9 (CRISPR/Cas9); and by short hairpin (sh)RNA-mediated silencing of transcribed messenger (m)RNA.

**Figure 2:**
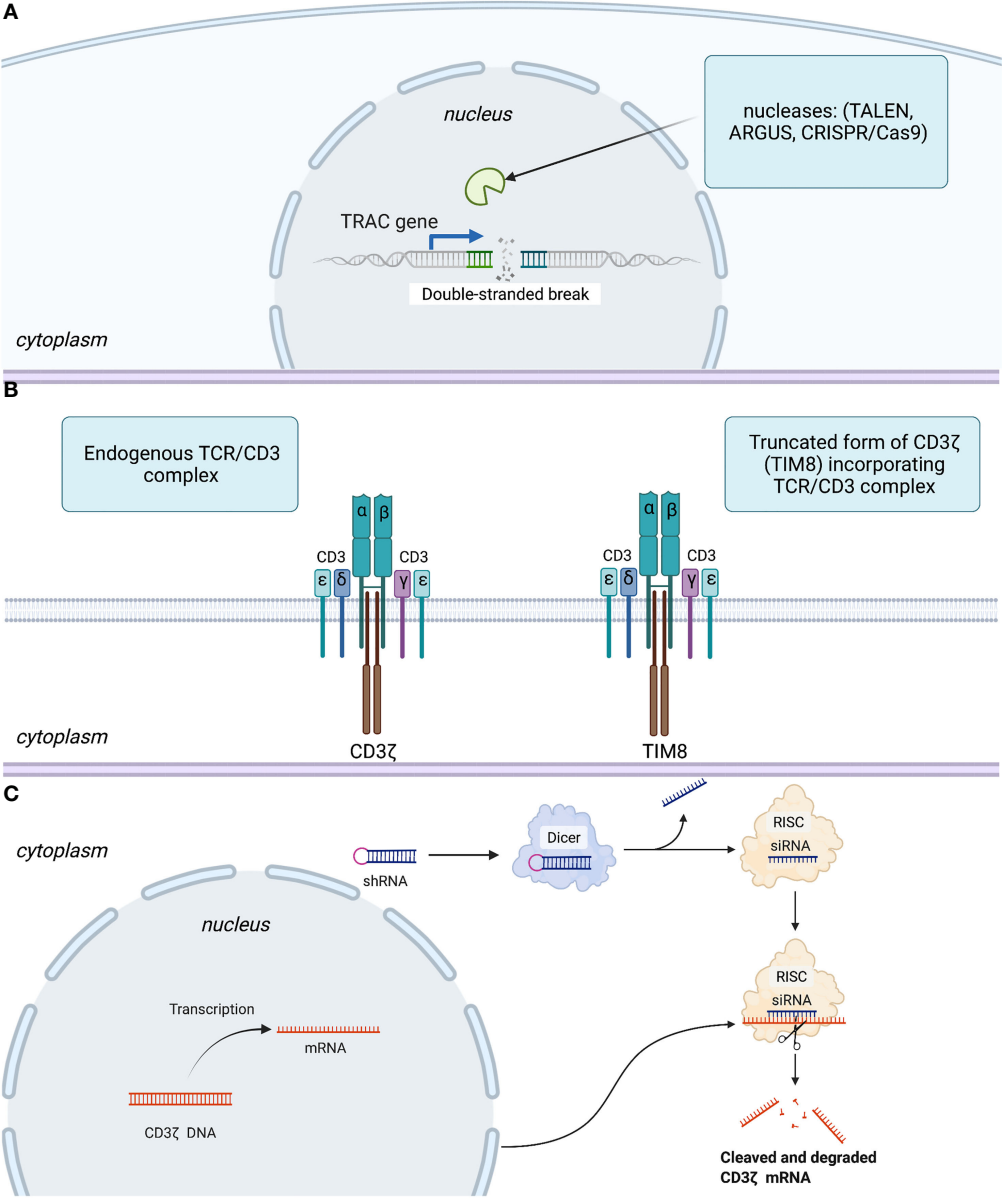
Three basic strategies to disrupt expression or signaling of TCRs. **(A)** Abolishing TCR expression by introduction of a double-stranded break in *TRAC gene.* ***(*B*)*** Interfering with TCR signaling using a competitive inhibitor of CD3ζ-TIM8. **(C)** Leveraging an RNA interference regulatory mechanism to silence mRNA coding for the CD3ζ component of the TCR. More specifically, RNase III endonuclease (Dicer) cuts the loop of the introduced shRNA homologous to the target within the CD3ζ genome. Furthermore, a guide strand (siRNA) is incorporated in the RNA-induced silencing complex (RISC), with subsequent transcriptional silencing of the target gene.

For instance, the very first technology utilized to remove αβTCR expression was based on TALENs targeting the T cell receptor alpha chain TRAC gene (**Figure 2A**), and efficiency of TCR elimination of 78% was recorded (45). The CRISPR/Cas9 efficiency of TCR KO was estimated to be 70% by Eyquem and colleagues (46). The use of zinc finger nucleases (ZFNs) to disrupt TCRs was first reported by Torikai and coworkers. Cells with anti-CD19 CAR were electroporated with ZFN mRNA targeting TCR alpha constant (*TRAC*) and *TRBC*, which abolished TCR signaling in 60 and 20% of cells, respectively (47). To generate their allogeneic CAR-T cell product PBCAR019 (particularly *via* insertion of the gene that encodes an anti-CD19 CAR into the *TRAC* locus), Precision BioSciences (Durham, NC, USA) used the ARCUS platform based on the engineered I-CreI homing endonuclease with the subsequent step of elimination of residual TCR+ cells. The engineered I-CreI homing endonuclease has shown 60% efficiency for TCR elimination previously (48).

Gilham and colleagues developed an approach leveraging a truncated dominant-negative CD3ζ protein (TIM). This protein acts as a competitive inhibitor to the component of TCR-CD3ζ, thereby interfering with TCR signaling (**Figure 2B**) and lowering the risk of GvHD (49). This approach was also used to disrupt TCRs in CYAD-101-CAR-T cells that target NKG2D. *In vivo* models assessing the efficiency of TCR inhibition showed a reduction in CD3-mediated stimulation and cytokine secretion in TIM-transduced T cells as well as no GvHD in mice in contrast with that in alloreactive control cells.

#### 2.1.2 Disruption of MHC-I Molecules

To increase the persistence of allogeneic CAR-T cells, the genetic abrogation of key mediators of immune rejection, MHC-I molecules (50), can be considered (**Figure 1**). This is best achieved by the disruption of a gene encoding β-microglobulin. Toricai et al. reported that transient expression of ZFNs specific for β-microglobulin resulted in the elimination of human leukocyte antigen (HLA)-A expression in ≤52% of the T-cell population (51). With a subsequent single round of depletion of HLA-A+ cells with antibody-coated paramagnetic beads, the fraction of HLA-A− cells increased up to 95%. Due to concern that cells without MHC-I molecules could be eliminated by host NK cells, Toricai et al. validated the approach to prevent NK cell-mediated cytotoxicity against MHC-I− allogeneic CAR-T cells. They ascertained that recognition by NK cells could be circumvented by enforced expression of HLA-E or HLA-G (51). Recently, it was shown that genetic abrogation of TCRs, as well as MHC-I (β2-microglobulin KO) and MHC-II (CIITA KO) molecules, ensured prolonged persistence of allogeneic CAR-T cells compared with that in cells with only TCR KO and MHC-I KO in a mouse model of cancer (52). Among allogeneic CAR-T cells products that include the disruption of the *β2m* locus alone with abrogation of TCR expression only CTX110 is at the clinical-trial stage (NCT04035434).

#### 2.1.3 Depletion of αβTCR+ Cells

None of the approaches described above remove 100% TCRαβ from a cell population. Hence, additional depletion of residual TCRαβ+ cells is an essential part of the manufacture of allogeneic CAR-T cells which, to a large extent, determines the absence of GvHD and therapy efficacy. The most advanced approach for eliminating TCRαβ+ cells is based on the CliniMACS™ device, which is described in detail elsewhere (53). In short, after introducing the CAR and TCR elimination, T cells are incubated first with biotinylated anti-TCRαβ antibodies followed by incubation with an anti-biotin antibody conjugated to magnetic microbeads. Thereafter, residual TCR+ cells are depleted by CliniMACS. Schumm et al. reported a median 0.00097% of residual TCRαβ cells after depletion using CliniMACS (53). Qasim et al. determined 0.7% of cells with detectable cell-surface TCRs after magnetic bead-mediated depletion (54).

Alexandre et al. investigated whether the transient expression of CARs targeting CD3 *via* mRNA electroporation would result in depletion of residual TCRαβ+ cells (55). To accomplish this goal, the authors first electroporated mRNA that encoded a TALEN targeting *TRAC*, followed by electroporation of an anti-CD3 CAR 49 h later. They observed that the minimum residual CD3+ TCRαβ+ population was 0.25%, which is comparable with results (0.7%) obtained with magnetic bead-mediated depletion of residual TCRαβ+ cells (54).

#### 2.1.4 Possible Genotoxicity of Gene-Editing Tools

If elucidating different approaches of genome editing, one should consider possible off-target events such as insertions, deletions, and chromosomal translocations (56). TALENs and ZFNs are dependent upon DNA–protein interactions, which are highly specific (57). In contrast, CRISPR/Cas9 is reliant on RNA–DNA interactions, which permit some mismatches, and therefore, lead to an increased risk of off-target events (58). However, Stadtmauer et al. reported chromosomal translocations and rearrangements after gene editing with CRISPR-Cas9 in a comparable percentage of cells (4%) as with gene editing using TALENs (59). They also mentioned that the efficiency and number of side effects using CRISPR/Cas9 correlated with a particular single guide (sg)RNA. Thus, optimization in preclinical evaluations *via* an accurate selection of suitable sgRNAs for gene targeting was possible. Hence, off-target events should be monitored *via* i*n silico* prediction with subsequent next-generation sequencing. Furthermore, we could speculate that, because the number of off-target events correlates with the number of gene edits, reducing the latter during the manufacture of CAR-T cells by insertion of a CAR directly into *TRAC* or gene silencing *via* shRNA could be essential to mitigate possible genotoxicity.

Stadtmauer et al. reported their experience with CRISPR/Cas9 to enhance antitumor activity in patients receiving autologous rather than allogeneic T cells engineered by lentiviral transduction to express cancer-specific (NY-ESO-1) TCRs (59). That report is interesting in terms of assessment of safety and possible genotoxicity. In particular, CRISPR/Cas9 was used to disrupt two genes encoding endogenous TCR chains (*TRAC* and *TRBC*), as well as programmed cell death protein 1. They reported chromosomal translocations and rearrangements (that declined *in vivo* and were at the limit of detection or not detected depending on the patient 30–170 days after infusion) in 4% of cells, which is similar to that employing genetic editing using TALENs (54). Off-target mutations were identified for *TRAC* sgRNA in chloride intracellular channel-2 (not expressed in T cells) and for *TRBC* sgRNA in transcriptional regulator ZNF609 and LINC00377 (long intergenic non-protein-coding RNA) (59). They mentioned more durable persistence of CRISPR-Cas9-edited T cells (up to 9 months) in contrast with cells that retained expression of the endogenous TCR and PD-1. A more recent single case of chromosomal abnormality was reported by Allogene Therapeutics (San Francisco, CA, USA) that was followed by a clinical hold of phase 2 ALLO-501A trial by the Food and Drug Administration (FDA) (60). The manufacturing process of these allogeneic CAR-T cells includes TALEN-mediated KO of TRAC and CD52 genes. As of October 7, 2021, the investigation is underway to clarify the potential relationship to gene editing and estimate the evidence of possible clonal expansion (60).

### 2.2 CAR-T Cells Based on Virus-Specific T Cells

Beyond genome-editing methods, one of the strategies to mitigate GvHD is to use CAR-T cells based on virus-specific T cells that combine the profound anti-tumor activity of the CAR and reduced risk of GvHD. GvHD risk corresponds to TCR diversity, therefore GvHD absence after infusion of virus-specific memory T cells is likely to be due to the restricted *repertoire* of TCRs in virus-specific memory T cells (61). For instance, the peptide GLCTLVAML is one of the most immunogenic T cell targets derived from the Epstein–Barr virus (EBV). Hence, T cell subsets with TCRα and β chains that specifically recognize this antigen on MHC complexes could be used in allogeneic settings without additional gene-editing steps to disrupt TCR expression. One of the possible *caveats* of this strategy could be the initial low numbers of virus-specific memory T cells. In peripheral blood during latent infection with EBV, T cells specific for this peptide constitute 0.5–2.2% of total CD8+ T cells (62).

### 2.3 γδ T Cells

Donor-derived αβ T cells recognize antigen bound to MHC molecules. Donor-derived γδ T cells recognize multiple tumor antigens by utilizing their innate receptors in an MHC molecule independent manner, and could be applied in an allogeneic setting without TCR elimination. Moreover, upon activation, they can, in turn, exert a further adaptive immune response by facilitating the function of other immune cells. Rozenbaum et al. investigated the possible use of donor-derived γδ T cells as the carriers of the CAR for the production of allogeneic CAR-T cells (63). They hypothesized that, in addition to functioning across MHC molecule-barriers without causing GvHD, γδ T cells could overcome the major issue of therapy using CAR-T cells: the loss of antigen on cancer cells. They reported encouraging results showing that, in addition to *in vivo* activity against tumor CD19+ cells, γδ CAR-T cells utilizing various surface receptors exhibited *in vitro* activity against CD19− clones (63). The Vγ9Vδ2 T-cell subset prevailing among γδ-T cells constitutes 2–4% of T cells in peripheral blood. Efficient expansion of this cell subset has been devised (64, 65). A phase-I clinical trial in 132 patients with late-stage cancer showed the feasibility and clinical safety of allogeneic Vγ9Vδ2 T cells (65). Hence, γδ T cells could be candidates for cellular tumor immunotherapy for metastatic and progressive solid malignancies that can address the tumor microenvironment.

### 2.4 Induced Pluripotent Stem Cells (iPSCs) as a Source for Allogeneic CAR-T Cells

Another option validated by Themeli et al. relies on the production of CAR-T cells from iPSCs (66). Briefly, the authors generated iPSCs from peripheral-blood T lymphocytes by transduction with retroviral vectors encoding reprogramming factors. Thereafter, multiple iPSC clones were screened to select the clone that was subsequently transduced with a lentiviral vector encoding an anti-CD19 CAR. Upon differentiation into a T-lymphoid lineage, the authors detected TCRαβ+ cells harboring the same rearrangements in TCRβ and γ chains as in the parental clone. The CAR-T cells of this origin are generated from one clonal pluripotent cell line, so we can conclude that they are phenotypically defined. To better describe the phenotype of iPSC-derived CAR-T cells, the authors turned to microarray analysis of gene expression and uncovered that the CAR-T cells generated from iPSCs resembled those in peripheral-blood γδ T cells (66). The authors also pointed out lower levels of CAR expression and shorter survival in an immunodeficient xenograft mouse model than those in CAR-T cells derived from the TCRαβ subset in peripheral blood (66). By contrast, more recent *in vivo* results in a disseminated xenograft model of lymphoblastic leukemia of FT819 (an anti-CD19 CAR-T-cell product derived from a clonal engineered iPSC line by insertion of genes encoding for novel 1XX CAR into the *TRAC* locus) showed enhanced clearance of tumor as compared with that in control anti-CD19 cells (67). Signal transduction by TCRs is initiated by phosphorylation of conserved immunoreceptor tyrosine-based activation motifs (68), and strong activation of T cells can drive exhaustion (69). Feucht et al. investigated whether the impaired redundancy of CD28 and CD3ζ signaling enhanced the therapeutic properties of CAR-T cells. They estimated that mutation in the 1XX tyrosine residue that impedes phosphorylation and downstream signaling increases persistence and extends the effector function of T cells (70). TFT819 was generated from a single clonal line with the bi-allelic disruption of TCRs with a minimum likelihood of GvHD. Functional assessment of FT819 showed potent cytolytic activity against leukemia and lymphoma lines and the inability to produce GvHD (67). However, the efficacy of FT819 warrants further clinical investigation that is being initiated by Fate Therapeutics (La Jolla, CA, USA) for patients with relapsed/refractory (r/r) B-cell malignancies (71). Overall iPSCs derived from CAR-T cells, despite some limitations, hold the potential of uniform and mass-produced CAR-T cells.

### 2.5 NK-92 Cell Line

Given the success of therapies using CAR-T cells for hematological malignancies, many researchers have sought to develop CAR-engineered NK cells. In contrast to CAR-T cells, CAR-NK cells cause minimal CRS or ICANS in autologous settings (72). However, there are technical challenges to obtain them because NK cells represent only ~10% of lymphocytes. In addition, in autologous settings, the function of NK cells can be impaired in patients with malignant disorders. Specifically, tumors employ upregulation of inhibitory ligands such as MHC-I molecules (HLA-G, HLA-E, and HLA-ABC) (73). Similarly, an immunosuppressive cancer microenvironment comprising regulatory T cells and myeloid-derived suppressor cells could decrease expression of activating receptors such as NKG2D and enhance expression of inhibitory receptors (NKG2A), thereby restricting the cytotoxicity of NK cells (74). Furthermore, autologous NK cells are functionally silenced upon encountering a self-MHC antigen. Conversely, blood-derived NK cells can carry the risk of GvHD in allogeneic settings because they may contain contaminating T cells (75). In this context, a clonal immortalized cell line from a patient with NK-cell lymphoma (NK-92) that could be expanded in the presence of IL-2 appears as a valuable alternative.

There are three main reasons why CAR-expressing NK cell lines such as NK-92 are of interest as allogeneic effectors for cell therapy.

- All cells used in the preparation originate from a single cell, so their properties are strictly determined and there is no product heterogeneity. In this regard, standardization is also simplified;
- There are no restrictions on the scale of cell modification. For example, multiple sequential transductions or rounds of genomic editing can be carried out to obtain a product with desired properties;
- NK-92 cells can be produced in any volume, up to multi-ton bioreactors, so this reduces (by several orders of magnitude) the cost of production significantly (76).

However, NK-92 are cancer cells, so they must be lethally irradiated before being injected into a patient to eliminate the chance of their engraftment or development of NK lymphoma. The latter, and the fact that the irradiated cells appear to be eliminated rapidly by the patient’s immune system, raise serious concerns about the feasibility of using CAR-NK-92 cells (77).

## 3 Translation of Allogeneic CAR-T Cells Into Clinical Use

The sources of allogeneic CAR-T cells described above are summarized in **Table 1**. This section focuses on products pioneering the technology of allogeneic CAR-T cells in the clinic.

**Table 1:** Allogeneic CAR-T cells sources.

CAR-T cells source	Therapy target	Necessity of gene editing
1. αβ T cells subsets	PBCAR0191 (CD19)	Insertion of CD19- specific CAR into the TRAC locus using versatile genome editing 785 platform ARGUS
	ALLO-715 (CD19)	TALEN-mediated CD52 and TRAC gene knockout
	ALLO-715 (BCMA)	TALEN-mediated CD52 and TRAC gene knockout
	UCART19 (CD19)	TALEN-mediated CD52 and TRAC gene knockout
	CTX110 (CD19)	CRISPR/Cas9 mediated insertion of CD19 CAR into TRAC locus and disruption of the *β2m* locus
	CYAD-101 (NKGD2D)	Gene editing of TCR is not needed due signaling inhibition (TIM)
	CYAD-211 (BCMA)	shRNA-mediated silencing of TCR signal is used
2. γδ T-cells subset	Kiromic announces submission of applications for PD1 γδ T cells	Gene editing or TCR is not needed due the absence of αβTCR
	CAR-T cell Therapy with the FDA	
3. Virus specific memory T cells	ATA3219 (CD19)	Gene editing of TCR is not needed due restricted repertoire
4. Induced pluripotent stem cells	FT819 (CD19)	CAR targeting CD19 inserted into the TRAC locus *via* CRISPR/Cas9

### 3.1 ALLO-715 Anti-BCMA CAR-T Cells (NCT04093596 Trial)

Allogene Therapeutics reported the results of a study on ALLO-715 anti-BCMA CAR-T cells. This was a phase-1 clinical trial (NCT04093596) in adults with r/r multiple myeloma who had ≥3 prior lines of therapy and were refractory to the last treatment line. As of October 2020, 31 patients had enrolled in the safety population. The efficacy population comprised 26 patients across four dosing levels of cells (40, 160, 320, and 480 × 106 CARs), with a median follow-up of 3.2 months (78). The CAR-T-cell receptor of ALLO-715 includes a single-chain variable anti-BCMA fragment with a 4-1BB costimulatory domain. To prevent graft rejection and allow for selective lymphodepletion without affecting ALLO-715 CAR-T cells, KOs of *CD52* and *TRAC* were introduced (79). Patients received lymphodepletion consisting of fludarabine plus cyclophosphamide and anti-CD52 antibody ALLO-647 in a set of different dosing regimens (**Table 2**). The overall response rate (ORR) across all dosing cohorts and lymphodepletion regimens among 26 patients evaluated for efficacy was 42% (11 patients) (78). The superior anti-cancer activity was observed among the 10 patients treated with 320 × 106 cells (dose level 3) of ALLO-715. For this cohort, the ORR was 60% (6/10 cases), which included a very good partial-plus response in four patients (40%).

**Table 2:** Conditioning regimes administered in clinical trials of autologous and allogeneic CAR-T products.

Product and corresponding clinical trial	Conditioning regimen
501 ALPHA (ALLO-501)	Fludarabine 30 mg/m^2^/day and cyclophosphamide 300 mg/m^2^/day given on 3 days with ALLO-647 (from 13 to 30 mg daily given for 3 days). The starting of lymphodepletion 5 days before the infusion ALLO-501.
UNIVERSAL (ALLO-715)	Fludarabine 30 mg/m^2^/day and cyclophosphamide 300 mg/m2/day given on the fifth, fourth, and third days before infusion ALLO-715 with ALLO-647 (13–30 mg × 3 days) or cyclophosphamide (300 mg/m^2^/day) given on the fifth, fourth, and third days before infusion ALLO-715 with ALLO-647 (13–30 mg × 3 days).
CARBON (CTX)	Fludarabine 30 mg/m^2^ and cyclophosphamide 500 mg/m^2^ given daily on the third, second, and first days before infusion CTX110. Infusion of CTX110 after completion of lymphodepleting chemotherapy.
PBCAR0191 (PBCAR0191)	Fludarabine 30 mg/m^2^/day and cyclophosphamide 500 mg/m^2^/day given on 3 days or fludarabine 30 mg/m^2^/day for 4 days and cyclophosphamide 1,000 mg/m^2^/day for 3 days. Infusion of PBCAR0191 after completion of lymphodepleting chemotherapy.
CARCIK (CARCIK—CD19)	Fludarabine 30 mg/m^2^/day × 4 days and cyclophosphamide 500 mg/m^2^/day × 2 days starting with the first dose of fludarabine. Infusion of CARCIK—CD19 from 2 to 14 days after completion of lymphodepleting chemotherapy.
PALL (UCART19)	Combining cyclophosphamide 60 mg/kg/day for 2 days, fludarabine 30 mg/m^2^/day for 5 days and alemtuzumab 0.2 mg/kg/day for 5 days starts during the week preceding UCART19 infusion (from Days 7 to 1).
CALM (UCART19)	Combining cyclophosphamide (1,500 mg/m^2^) and fludarabine (90 mg/m^2^) without (FC) or with alemtuzumab (FCA) (1 mg/kg) was administered one week before UCART19 infusion.
ELIANA (KYMRIAH): Pediatric and Young Adult Relapsed or Refractory (r/r) B cell Acute	Fludarabine 30 mg/m^2^ IV daily for 4 days and cyclophosphamide 500 mg/m^2^ IV daily for 2 days starting with the first dose of fludarabine.
Lymphoblastic Leukemia (ALL):	
ELIANA (KYMRIAH): Adult	Fludarabine 25 mg/m^2^ daily for 3 days and cyclophosphamide 250 mg/m^2^ IV daily for 3 days starting with the first dose of fludarabine. Infuse KYMRIAH from 2 to 14 days after completion of the lymphodepleting chemotherapy.
Relapsed or Refractory (r/r) Diffuse	
Large B cell lymphoma (DLBCL)	
ZUMA-5 (Yescarta)	Fludarabine 30 mg/m^2^/day and cyclophosphamide 500 mg/m^2^/day given on the fifth, fourth, and third days before infusion YESCARTA
TRANSCEND NHL 001	Fludarabine 30 mg/m^2^/day and cyclophosphamide 300 mg/m^2^/day for 3 days. Infuse BREYANZI from 2 to 7 days after completion of lymphodepleting chemotherapy.
(BREYANZI; lisocabtagene	
maraleucel; liso-cel)	
ZUMA-2 (TECARTUS; Brexucabtagene Autoleucel;KTEX19): Mantle Cell Lymphoma.	Fludarabine 30 mg/m^2^ daily for 3 days, and cyclophosphamide 500 mg/m^2^ daily for 3 days.
ZUMA-2 (TECARTUS; Brexucabtagene Autoleucel;KTEX19): Acute lymphoblastic leukemia.	Fludarabine 25 mg/m^2^ iv on the fourth, third, and second days and cyclophosphamide 900 mg/m^2^ on the second day before infusion of TECARTUS.

TRANSCEND NHL 001 clinical trial (80), ZUMA-2 clinical trial (81), ELIANA clinical trial (10), ZUMA-5 clinical trial (22).

CRS was reported in 45% (14/31) patients. One grade-5 episode in a patient who developed non-neutropenic fever and multifocal pneumonia one day after ALLO-715 infusion led to respiratory failure and death. The authors considered this episode to be related to progressive myeloma and the conditioning regimen with cyclophosphamide and ALLO-647 (79). The authors reported that infectious diseases developed during therapy in 42% (13/31) patients, including grade 3 infections in 13% of patients. Cases of GvHD or ICANS were not observed (**Table 3**).

**Table 3:** Efficacy and adverse events associated with allogeneic and autologous CAR T cell therapy.

Product	Allogenic CAR-T						Autologous CAR-T	
	ALLO-715	ALLO-501	UCART19	CTX110	PBCAR0191	CARCIKCD19	Kymriah	Yescarta
Clinical trial	UNIVERS AL (NCT04093596)	ALLO-501 ALPHA NCT03939026	CALM/PALL NCT02746952/NCT02808442	CARBON NCT04035434	PBCAR0191 NCT036660 00	CARCIK NCT03389035	ELIANA NCT02435849	ZUMA-5 NCT03105336
Number of patients that received	31 patients	41 patients	21 patients	11 patients	27 patients	13 patients	75 patients	146 patients
CAR-T								
Disease	Relapsed/Refractory Multiple Myeloma	Relapsed/Refractory large B cell lymphoma and follicular lymphoma	Refractory or relapsed B cell ALL	Refractory or relapsed non-Hodgkin lymphoma Refractory or relapsed B cell	Refractory or relapsed non-Hodgkin lymphoma	Relapsed or refractory adult and pediatric B cell precursor ALL after HSCT	Refractor y or relapsed B cell	Relapsed or Refractor y large B-cell lymphoma
				ALL	Refractory or relapsed B cell ALL		ALL	
ECOG	0–1	0–1	<2	0–1	0–1	N/D	N/D	0–1
Adverse Events of Interest, pts	31	41	21	11	27	13	75	146
CRS	Gr 1–2: 14	Gr 1–2: 11	Gr 1–2 16	Gr 1–2:	≥3 Gr absent	Gr 1–2: 3	Gr 1–2:	119/146
	(45%) ≥Gr 3 absent	(27%) ≥Gr 3 absent	(76%) ≥3 Gr 3 3(16%)	Gr 3 absent		(23%) ≥Gr 3 absent	23 (31%) ≥Gr 3:35 (46%)	(81,5%) ≥Gr 3:7%
ICANS	absent	Gr 3 1(2%)	Gr 1–2: 8	Gr 1–2: 1 (9%)	≥ Gr 3 single case	absent	Gr 1–2: 20	87/146 (59,6%) including ≥Gr3:
			(38%) ≥Gr3 absent				(27%) Gr3: 10 (13%)	19%
Infections	13 (42%)	25 (61%)	13 (62%)	3 (27%)	4/18 (22%)	4(30%)	32 (43%)	16%
					NHL			
GvHD	absent	absent	2 (10%)	absent	absent	absent	–	–
			Skin 1Gr					
Efficacy, pts	26	32	21	11	27	13	75	104
*ORR, n (%)	11(42%)	24 (75%)	–	4 (36%)	15 (55%)	–	81%	92%
*CR, n (%)	**–**	16 (50%)	14(67%)	4 (36%)	10 (37%)	8 (61.5%)	60%	76%
			82% (AL)		75% (IL)			

^*^ORR and CR is shown for patients among all dosing cohorts and lymphodepletion regimes according to recently published results of allogeneic CAR-T cells trials described above and results of clinical trials of autologous products Kymriah (10) and Yescarta (22).

AL, patients with alemtuzumab-containing lymphodepletion.

IL, (patients with relapsed or refractory non-Hodgkin lymphoma who received enhanced lymphodepletion regimen).

ND, not detected.

### 3.2 ALLO-501 Anti-CD19 CAR-T Cells (NCT03939026 Trial)

According to a recent report, ALLO-501 (Allogene Therapeutics) showed positive results in a trial (NCT03939026) for patients with relapsed/refractory non-Hodgkin lymphoma (r/r NHL) who had ≥3 prior lines of therapy (82). As of April 19, 2021, 41 patients had received ALLO-501, 41 patients had enrolled in the safety population and the efficacy population included 32 patients across three dosing levels of cells (40, 120 and 360 × 106 CARs). The ALLO-501 CAR-T receptor is based on murine CD19 specific (4G7) scFv. In addition, TALEN-mediated KO of TRAC and CD52 genes were introduced.

Therapy comprised prior lymphodepletion including fludarabine plus cyclophosphamide (**Table 2**) with ALLO-647 and infusion of ALLO-501 CAR-T cells. The ORR was 75% (24/32 patients), with 50% (16/32) cases having a complete response (CR).

The authors reported mild-to-moderate CRS in 11 (27%) patients, one (2%) case of grade-3 neurotoxicity, and no GvHD among enrolled patients. The prevalence of infection was 61% (25/41 cases), which was similar to the prevalence observed in trials using autologous CAR-T cells (82).

### 3.3 UCART19 Anti-CD19 CAR-T Cells (PALL and CALM Trials)

Another CAR-T cell product based on lentiviral transduction of CAR19 and the use of TRAC/CD52 specific TALENs is UCART19 (54). TALENs are used to introduce KOs in genes encoding the α constant chain of TCRs and CD52 to minimize GvHD risk and to confer resistance to the anti-CD52 monoclonal antibody alemtuzumab (54). Residual TCR+ cells were removed by magnetic beads [CliniMACS (53)] and only 0.7% of cells had detectable cell-surface TCRs. The vector also incorporates a 2A peptide-linked sort/suicide gene (RQR8), which includes CD34 and CD20 epitopes for cell enrichment, and rituximab is used for *in vivo* depletion in case of adverse effects (83). Unexpectedly, RQR8 expression was further detected by flow cytometry in only 19.9% of cells, despite linked transcription and translation through a self-cleaving 2A peptide configuration of RQR8 with highly expressed (85% of cells) CAR19 (54). More than 64% of cells also exhibited a CD52− phenotype. The authors revealed a high representation of the CD8-phenotype subset together with “naive-like” phenotypes. If using gene-editing nucleases such as TALENs, one should consider possible off-target events (nonhomologous end-joining, insertions, deletions). Using next-generation sequencing, the authors detected <0.18% off-target events at 15 *in silico*-predicted off-target TALEN sites.

The efficacy of therapy with UCART19 was first evaluated in two infants with relapsed B cell acute lymphoblastic leukemia. The complete protocol of this therapy is described elsewhere (54). Summing up, before UCART19 infusion, lymphodepletion (fludarabine, cyclophosphamide, and alemtuzumab) was administered (54). The authors reported no infusion-related toxicities and no evidence of CRS. Grade 2 skin GvHD was observed by histology in one patient at 9 weeks and resolved after corticosteroids treatment. Finally, to correct aplasia and accelerate reconstitution, the TCRαβ-depleted allograft from the original mismatched unrelated donor was administered. Patients were in complete remission after eradication of UCART19 and transplantation.

The safety and efficacy of UCART19 were further evaluated in the PALL trial in seven children and in the CALM trial in 14 adults. Patients had to have evidence of CD19+ B cell acute lymphoblastic leukemia with >5% blasts in bone marrow or a minimal residual disease of 1 × 10^−3^ cells as assessed by flow cytometry or quantitative polymerase chain reaction. Before UCART19 infusion, all the patients underwent lymphodepletion: 17 patients (81%) with fludarabine, cyclophosphamide, and alemtuzumab, and four (19%) with fludarabine and cyclophosphamide (**Table 2**).

Children in the PALL trial received UCART19 (1.1–2.3 × 10^6^ per kg) and the CALM trial included a dose-escalating phase (6 × 10^6^ cells, 6–8 × 10^7^ cells, or 1.8–2.4 × 10^8^ cells) (84). The ORR was 67% (14/21) and 82% (14/17) for patients receiving alemtuzumab-containing lymphodepletion (84). Ten (71%) of the 14 patients achieved a complete response (CR) and proceeded to allogeneic hematopoietic stem cell transplantation (HSCT). The authors reported that, at the data cutoff of August 2019, 10 (71%) of the 14 patients who achieved a CR or CR with incomplete hematologic recovery (including patients who underwent SCT) had subsequently relapsed or died. Progression free survival at 6 months was 27% (95%CI 10–47). All but one of these relapsing patients were CD19+.

The adverse effects observed with UCART19 seem similar to those reported for autologous antiCD19 CAR-T cells. CRS was the most common adverse event associated with the UCART19 treatment (91% of patients). CRS of grade ≥3 was documented in three patients. Other adverse events were neurotoxicity of grade 1 in seven patients and grade 2 in one patient that lasted at a median duration of 3 days and did not require specific treatment. Only two patients (10%) developed grade 1 GvHD after UCART19 infusion. One death in the CALM trial reported as the dose-limiting toxicity of UCART19 was caused by neutropenic sepsis with grade 3 CRS, and the other death was caused by pulmonary hemorrhage occurring in the context of infection and grade 4 cytopenia. Although, reactivation of infection by cytomegalovirus, adenovirus and EBV was observed particularly in patients receiving high doses of alemtuzumab (anti-CD52 monoclonal antibody), omitting alemtuzumab abolished UCART19 expansion. Therefore, subsequently, the dose of alemtuzumab was reduced to prevent severe viral infections and enable UCART19 expansion.

Benjamin and colleagues (84) stated that grade 4 cytopenia in 32% of patients during therapy was probably associated with an intensive lymphodepletion regimen required to overcome HLA barriers (alemtuzumab in combination with fludarabine and cyclophosphamide). Of note, 3–6% of TALENedited UCART19 cells used in the trials had translocation-associated karyotype abnormalities with yet unrevealed adverse effects (84). Despite initial concern, residual infused TCR+ cells after expansion did not cause transfusion-associated GvHD during the CALM and PALL trials. Both could have been due to ablation of residual UCART19 cells before allogeneic HSCT (84).

### 3.4 CTX110 Anti-CD19 CAR-T Cells (NCT04035434 Trial)

UCART19 and other CAR-T cells use randomly integrating viruses to deliver genes encoding CAR constructs to T cell DNA. CRISPR Therapeutics (Cambridge, MA, USA) inserted a CAR construct precisely into the *TRAC* locus using CRISPR/Cas9. Hence, TCR KO and the introduction of the CAR are achieved in one step. CRISPR/Cas9 is also used to disrupt the *β2m* locus, thus eliminating the expression of MHC-1 molecules.

The results from CRISPR Therapeutics’ ongoing phase-1 CARBON trial (NCT04035434) evaluating the safety and efficacy of CTX110 in 11 patients with r/r NHL who had ≥2 prior lines of treatment have been announced. CTX110 targets CD19+ B-cell malignancies. Eleven patients were infused with CTX110 cells at four dose levels (30, 100, 300 or 600 × 10^6^) after lymphodepletion consisting of fludarabine and cyclophosphamide (**Table 2**). Among the patients who received 30–300 × 10^6^ CTX110 cells, the authors reported no cases of GvHD despite a high HLA mismatch between donors and patients, three cases of grade ≤2 CRS (30%) and one case of grade 2 ICANS (10%) (85). A patient who received 600 × 10^6^ CTX110 cells experienced grade 2 CRS, febrile neutropenia, and developed short-term memory loss and confusion (which later progressed to significant obtundation), reactivation of HHV-6 (Human Herpes Virus), and HHV-6 encephalitis (85). A complete response was achieved in 36% (four) of patients at 100, 300, and 600 × 106 CTX110 cells. At 300 × 106 CTX110 cells, two out of four patients had a complete response (85).

### 3.5 PBCAR0191 Anti-CD19 CAR-T Cells (NCT03666000 Trial)

Precision BioSciences makes the allogeneic product PBCAR0191. It is created by the insertion of a CD19-specific CAR into the *TRAC* locus using the versatile genome-editing platform ARCUS, which is based on I-CreI homing endonuclease (86). Then, cells undergo a D3 elimination step, followed by expansion and freezing (87). Preliminary data are from the phase-I study of PBCAR0191 cells from 27 patients (16 with r/r NHL, 11 with r/r B-cell acute lymphoblastic leukemia) who had ≥2 previous lines of treatment (88). PBCAR0191 treatment was undertaken at dose level 1 (3 × 105 cells), dose level 2 (1 × 10^6^ cells), dose level 3 (3 × 10^6^ cells), and a split dose level 4 (two doses at 3 × 10^6^ cells employed after standard lymphodepletion consisting of fludarabine plus cyclophosphamide). PBCAR0191 was also dosed in an enhanced lymphodepletion regimen consisting of PBCAR0191 at dose level 3 (3 × 10^6^ cells) or dose level 4 (two doses at 3 × 10^6^ cells plus fludarabine (30 mg/m2/day for 4 days) and cyclophosphamide (1,000 mg/m^2^/day for 3 days).

The ORR and complete response across all dosing cohorts and lymphodepletion regimens was 55% (15/27 cases) and 37% (10/27 cases), respectively. The authors reported an 83% ORR at day-28 or later for patients with NHL or B-cell acute lymphoblastic leukemia who received PBCAR0191 when coupled with enhanced lymphodepletion. On day-28 or later, 75% of patients with r/r NHL who received PBCAR0191 with enhanced lymphodepletion achieved a complete response, versus only 33% across dose level 2 (1 × 10^6^) and dose level 3 (3 × 10^6^ cells) using standard lymphodepletion (88). Hirayama et al. sought to identify the biomarkers associated with a complete response and progression-free survival in patients with aggressive B cell NHL after autologous anti-CD19 CAR-T cell therapy. Patients receiving high-intensity lymphodepletion had a higher probability of achieving a favorable cytokine profile (IL-7 and serum monocyte chemoattractant protein-1) that correlated with a better complete response and progression-free survival compared with that in patients receiving low-intensity lymphodepletion (89).

More recently, Shah et al. (90) published clinical trial results concerning patients cohort to whom the PBCAR0191 was dosed at level 3 (3 × 10^6^ cells) and coupled with enhanced lymphodepletion (fludarabine 30 mg/m^2^/day × 4 days plus cyclophosphamide 1,000 mg/m^2^/day × 3 days). Twenty one patients were enrolled, including 16 patients with NHL and 5 patients with B-ALL with measurable CD19+ R/R B-ALL or NHL disease after two or more prior treatment regimens. The authors reported profoundly improved PBCAR0191 kinetics compared to patients to whom standard lymphodepletion was administered. The treatment efficiency was assessed in 13 patients with NHL and in 5 subjects with ALL. The overall response was 83% (15/18) patients, including 85% (11/13) patients with NHL and 80% (4/5) ALL subjects, with 50% (12/18) cases having a complete response (CR/CRi) including 62% (8/13) patients with NHL and 80% (4/5) ALL subjects. Most adverse events were mild. The authors reported that ICANS Grade 3 was observed in one patient with NHL, grade 3 infections observed in 31% (5/16) patients with NHL, and 80% (4/5) patients with B-ALL. No evidence of GvHD was observed.

### 3.6 CYAD-101 (CYAD-211 Trials)

Celyad Oncology manufactures CYAD-101. This product combines a human full-length NKG2D receptor that binds eight different ligands expressed by cancer cells of different origins in an MHC molecule-independent fashion (91) and a TCR inhibitory peptide that interferes with signaling by the endogenous TCR. CYAD-101 was evaluated in the alloSHRINK phase-I study in patients with unresectable metastatic colorectal cancer (NCT03692429). After standard preconditioning chemotherapy (FOLFOX), 15 patients received one of three dose levels (1 × 10^8^, 3 × 10^8^ or 1×10^9^ cells per infusion). The authors reported no dose-limiting toxicity or GvHD. CYAD-101 at 1×10^9^ cells per injection post-FOLFOX chemotherapy was used. Out of 15 patients, two (13%) patients achieved a partial response and nine (60%) cases had stable disease (92).

In parallel, Celyad Oncology investigated (93) another approach to prevent GvHD by leveraging shRNA to silence the mRNA coding for the CD3ζ component of the TCR (**Figure 2C**). In particular, their new product, CYAD-211, which is designed to express anti-BCMA CAR and shRNA interfering with CD3ζ expression, is being evaluated in the phase-I IMMUNICY-1 trial (NCT04613557) for the treatment of patients with r/r multiple myeloma. The authors pointed out a high percentage of shRNA-CD3ζ knockdown comparable with that using CRISPRs targeting CD3ζ to inhibit TCR expression.

### 3.7 Cytokine-Induced Killer (CIK) Anti-CD19 Cells (NCT03389035 Trial)

Magnani et al. proposed a nonviral engineering of allogeneic CAR-T cells based on a “Sleeping Beauty” transposon system to produce CIK cells with CARs (94). According to the CIK-cell protocol, cells were stimulated to differentiate to a subpopulation of memory T cells (95). Cells were derived from four matched unrelated donors, six haploidentical donors and three siblings with identical HLAs. During multicenter clinical studies (NCT03389035), CARCIK-CD19 cells were administered to 13 patients with B cell acute lymphoblastic leukemia who had relapsed after HSCT. The authors reported no cases of ICANS or GvHD even in patients who experienced GvHD after initial HSCT (**Table 3**). The only severe adverse events were two cases of grade 1 and grade 2 CRS in patients receiving the highest dose. A complete response was noted in 61.5% of patients whereas, among the six patients receiving the two highest doses, a complete response was noted in 85.7% of cases. The authors pointed out that the absence of manageable GvHD after the infusion of CIK cells was associated with the acquisition of MHC molecule-independent NK-like cytotoxicity during stimulation with interferon-γ, CD3, and differentiation in the presence of IL-2 (96). The insertions of Sleeping Beauty did not appear to trigger clonal dominance, while in rare cases, the insertion of *CAR* with a lentiviral vector might alter T-cell regulatory pathways due to preferable integration into highly expressed genes triggering clonal expansion [e.g., vector insertion within the CBL oncogene (97) and disruption of the TET2 allele (98)].

### 3.8 ATA188 and the NCT03283826 Trial

Prockop et al. revealed that EBV-targeted T cells demonstrated a favorable safety and limited risks of GvHD or CRS in 46 recipients with rituximab-refractory EBV-associated lymphoma (99). Curran et al. reported a complete response in 70% (7/10 patients with r/r B-cell malignancies) and an absence of ICANS, CRS, or GvHD above grade 2 after treatment with a CD19-specific CAR developed by transducing EBV-specific donor cells (100).

Preliminary results utilizing EBV-specific subsets of T cells were demonstrated by Atara Biotherapeutics in a phase-I trial using ATA188 (NCT03283826). GvHD or CRS after the infusion of ATA188 cells was not documented. The authors reported that ATA188 was well-tolerated in patients with progressive multiple sclerosis, and dose-limiting toxicities were not reported. ATA188 was manufactured from lymphocytes specific for the EBV antigens of an unrelated (but partially HLA-matched) donor. Atara Biotherapeutics also developed EBV-specific T cells with a CAR targeting CD19. Their product (Allo-EBV-CD19-CAR-T) expresses an anti-CD19 CAR and maintains the expression of the native EBV TCR (101). Allo-EBV-CD19-CAR-T demonstrates a robust killing of antigen+ cells, antigen-specific proliferation in the presence of EBV and CD19+ cells, an enriched central memory phenotype (with higher frequency expression of CD62L, CCR7, and CD45RO), and provides a framework for developing next-generation allogeneic CAR-T cells: ATA3219.

### 3.9 Vγ9Vδ2 T Cells

Xu et al. published the results of the clinical trials of allogeneic Vγ9Vδ2 T-cell therapy for 132 patients with late-stage malignant liver ((NCT03183232), lung (NCT03183219), pancreatic (NCT03183206) or breast (NCT03180437) cancer. The details of the protocol and results are described elsewhere (65). Significant adverse events (CRS, GvHD) were not reported after infusion of allogeneic Vγ9Vδ2 T cells.

### 3.10 NK Cells

NK cells are candidates for engineering allogeneic CAR-NK cells for cancer treatment. Liu et al. reported the results of phase-I and -II trials in which HLA-mismatched anti-CD19 CAR-NK cells derived from cord blood were administered to 11 patients with r/r NHL or chronic lymphocytic leukemia (102). NK cells were transduced with a retroviral vector encoding an anti-CD19 CAR, IL-15 and inducible caspase 9 (to trigger apoptosis of CAR-NK cells in case of unacceptable toxic effects). The authors reported a median of 0.01% contaminating CD3+ T cells in the final product and the absence of CRS, ICANS, and GvHD (102). The authors mentioned stable levels of the proinflammatory cytokines IL-6, interferon-γ, and TNF. All patients had reversible hematologic toxic events, mainly associated with lymphodepletion. At a median follow-up of 13.8 months, seven patients (64%) had a complete response.

In the sole completed CAR-NK-92 trial (CD33-specific for patients with acute myeloid leukemia), cells were injected thrice at doses 3 × 10^8^, 6 × 10^8^ and 1 × 10^9^ cells on days 1, 3, and 5, respectively (103). Of the three patients in the trials, two patients experienced mild pyrexia and one episode of pyrexia up to 40°C that resolved in 2 days. Tang et al. (103) reported only two cases of grade-I CRS and no cases of GvHD after the infusion of CD33-CAR NK-92 cells. Hence, CAR NK-92 cells (at least with this particular CAR) were as safe as the unmodified parental NK-92 cell line (104). Unfortunately, two patients relapsed and one had no response to treatment. CAR-T-cell therapy in patients with acute myeloid leukemia remains challenging because antigens (e.g., CD123) used as a target for CAR-T cells are also expressed in normal hematopoietic stem cells and myeloid cells, which can cause severe CRS, neurotoxicity and off-target events (105, 106). Therefore, in terms of side effects, NK-92 cells as CAR carriers appear to be safe, but cases of a complete response after their injection have not been described.

## 4 Discussion

Presently, all FDA-approved CAR-T cell therapies are based on the autologous T cells isolated from the patient. Clearly, the use of a CAR-T cell product derived from a healthy donor appears advantageous, given that it would be less dependent on the immune status of the patient, be more standardized and greatly expand the patient access to the therapy due to lower production costs. It must also be taken into account that upon rapid disease progression the time required for autologous CAR-T cell manufacturing becomes a critical factor. During this time, tumor burden increases which may translate into a lower survival rate. Furthermore, a fraction of patients may ultimately never receive the autologous CAR-T cell product of decent quality and therapeutically meaningful quantity. In contrast, allogeneic CAR-T cells produced from healthy donors may display significantly better cellular fitness at the time of infusion, and by default, such CAR-T cell products would be free of contaminating tumor cells, unlike in the autologous format. In this regard, similarly to BiTEs (107), allogeneic CAR-T cell products are “off-the-shelf” and combine the advantages of both platforms. Nonetheless, these formats are not exactly interchangeable, and each of them has its own niche. Depending on the clinical presentation, BiTEs, autologous CAR-T, and allogeneic CAR-T cell products can be successfully used consecutively and even target the same antigen.

**Figure 3:**
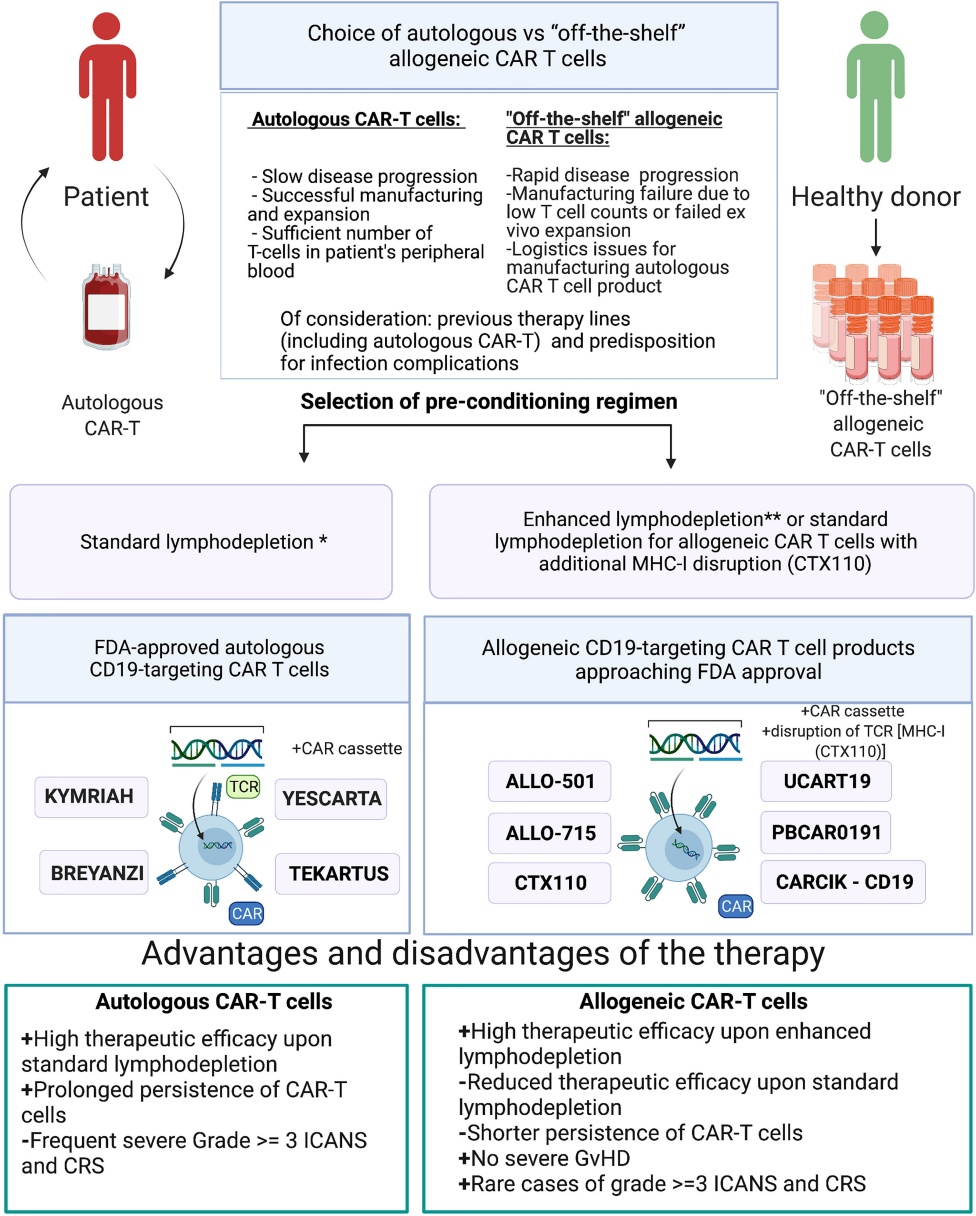
Choice of either autologous or allogeneic CAR-T cell therapy. *Standard lymphodepletion—conditioning regimen in which intermediate doses of chemotherapy drugs are applied. **Enhanced lymphodepletion—conditioning regimen in which high doses of chemotherapy drugs and/or additional biologic medications (monoclonal antibody, including allo-647) are applied.

There are several scenarios when allogeneic CAR-T cells can and should be used in place of or in combination with the autologous CAR-T cells: i) logistics issues preventing the manufacturing of the autologous product; ii) manufacturing failure, wherein the allogeneic product may substitute the autologous one without delaying the scheduled infusion; iii) infusion of the allogeneic CAR-T cell product when the autologous product fails to expand in the patient; iv) autologous CAR-T cell product cannot be manufactured upfront due to low T cell numbers in the patient and/or rapidly progressing disease; v) allogeneic CAR-T cell product serves as a bridge to hematopoietic stem cell transplantation by design; and vi) dose-adjusted infusion of allogeneic CAR-T cells with a low chance of engraftment to achieve progressive tumor de-bulking, thereby reducing the chance and magnitude of adverse side effects by the time of infusion of autologous CAR T cell product (**Figure 3**).

Allogeneic CAR-T cells may cause GvHD and may themselves be rejected by the immune system of the recipient. The frequency of such adverse effects is associated with the fine details of the manufacturing protocols and the procedures of how such cells are rendered universal to avoid allorejection. One avenue to avoid destruction of the allogeneic CAR-T cell product is to induce immunosuppression in the patient, but this must be finely balanced and closely monitored to reduce the risk of life-threatening infections. In most clinical trials of allogeneic CAR-T cells, GvHD never progressed beyond stage I, and only skin involvement was observed (**Table 3**). Therefore, we arrived into the conclusion that gene editing and subsequent cell-processing steps (depletion of TCR+ cells using magnetic beads) enable nearly complete elimination of residual TCRαβ+ cells (<1%) below the thresholds that might cause clinically significant GvHD. Therapy toxicity evaluation of the reviewed allogeneic CAR-T clinical trials revealed single cases of severe (≥3 grade) CRS and ICANS (**Table 3**), while more frequent development of severe (≥3 grade) CRS and ICANS was observed in patients who received autologous CAR-T therapy (clinical trials ELIANA, ZUMA-5). Higher doses of allogenic CAR-T cells and/or enhanced lymphodepletion were associated with higher efficacy of allogeneic CAR-T cells comparable with efficacy of autologous products (**Tables 2**, **3**). Under standard lymphodepletion, allogeneic CAR T cells had inferior efficacy and response rates compared to the autologous CAR-T cells, which is largely attributable to their lower persistence. This can be viewed as a surmountable issue. Furthermore, this can be considered as a safety advantage in the long run, particularly in the context of CAR T cells targeting the molecules present on the healthy tissues and organs. In fact, multiple autologous CAR T cell infusions have similarly been reported (e.g., NKG2D-CAR) as a means to counteract relatively short persistence. Finally, in the absence of contraindications enhancing the lymphodepletion regimen prior to CAR T cell infusion may ultimately obviate the need for such multiple infusions.

The limitation of this review is that data concerning clinical trials of ALLO-715, ALLO-501, CTX110, and PBCAR0191 are based on press releases, published by the corresponding companies. Notwithstanding this limitation, all studies showed the feasibility of administering allogeneic CAR-T cells and provide a path for more widespread and efficacious anticancer therapy.

## Author Contributions

All authors listed have made a substantial, direct, and intellectual contribution to the work and approved it for publication.

## Funding

This work was financially supported by the Ministry of Science and Higher Education of the Russian Federation (075-15-2020-901).

## Conflict of Interest

The authors declare that the research was conducted in the absence of any commercial or financial relationships that could be construed as a potential conflict of interest.

## Publisher’s Note

All claims expressed in this article are solely those of the authors and do not necessarily represent those of their affiliated organizations, or those of the publisher, the editors and the reviewers. Any product that may be evaluated in this article, or claim that may be made by its manufacturer, is not guaranteed or endorsed by the publisher.
